# The uptake of 3H-vincristine by a mouse carcinoma during a course of fractionated radiotherapy.

**DOI:** 10.1038/bjc.1989.276

**Published:** 1989-09

**Authors:** G. D. Zanelli, L. Rota, M. Trovo, E. Grigoletto, M. Roncadin

**Affiliations:** Clinical Research Centre, Harrow, Middlesex, UK.

## Abstract

The variations in uptake of 3H-vincristine sulphate, given as a bolus i.v. injection, by a transplantable murine tumour during a realistic course of fractionated daily gamma-radiation of 25 x 2.0 Gy have been investigated. Maximum levels of 3H in the tumours are found when the tracer is injected 4h after irradiation and the tumours are dissected out 1 h after injection. During the course of daily irradiation the pattern of uptake varies considerably but reproducibly. There are peaks of uptake after 7, 13 and 22 fractions of 2.0 Gy when the amount of 3H in the tumours is as much as three times that found in non-irradiated tumours. After 17-18 fractions, however, the tumour content of 3H is lower than that of non-irradiated tumours. The wave-like pattern of uptake could be due either to capillary occlusion brought about by radiation induced cellular swelling and oedema followed by re-opening of the capillaries during periods of decreased cellularity, or to some mechanism of recovery from radiation damage during the week-end rest period.


Br. J. Cancer (1989), 60, 310–314
© The Macmillan Press Ltd., 1989

### The uptake of $^3$H-vincristine by a mouse carcinoma during a course of fractionated radiotherapy

G.D. Zanelli[1], L. Rota[2], M. Trovo[3], E. Grigoletto[2] & M. Roncadin[3]

[1]*Clinical Research Centre, Watford Road, Harrow, Middlesex HA1 3UJ, UK;* [2]*Istituto Raphael, Calcinato, and* [3]*Radioterapia e Oncologia Medica, Ospedale Civile, Pordenone, Italy.*

**Summary** The variations in uptake of $^3$H-vincristine sulphate, given as a bolus i.v. injection, by a transplantable murine tumour during a realistic course of fractionated daily $\gamma$-radiation of $25 \times 2.0$ Gy have been investigated. Maximum levels of $^3$H in the tumours are found when the tracer is injected 4 h after irradiation and the tumours are dissected out 1 h after injection. During the course of daily irradiation the pattern of uptake varies considerably but reproducibly. There are peaks of uptake after 7, 13 and 22 fractions of 2.0 Gy when the amount of $^3$H in the tumours is as much as three times that found in non-irradiated tumours. After 17–18 fractions, however, the tumour content of $^3$H is lower than that of non-irradiated tumours. The wave-like pattern of uptake could be due either to capillary occlusion brought about by radiation induced cellular swelling and oedema followed by re-opening of the capillaries during periods of decreased cellularity, or to some mechanism of recovery from radiation damage during the week-end rest period.

The substantial number of recent publications on the subject indicate that the use of combined modality treatment (CMT; Howes, 1988) is increasing. In CMT, chemotherapeutic agents are given either before, during or after a course of fractionated radiotherapy.

The choice of the type of chemotherapeutic agent(s) to be administered and the timing of its administration are based or radiobiological principles as well as consideration of normal tissue damage and the general state of health of the patient (Steel, 1988; Brown, 1979; Tubiana *et al.*, 1985). Thus when oncologists speak of 'radiation–drug interaction' they mean the enhancement of, or protection from, the effects of radiation on normal and/or malignant tissues (Steel, 1979). Although these considerations are logical and valid there is one effect of radiation which is never taken into account but which may have significant implications for the success or failure of CMT: the effects of radiation on vascular integrity.

It is by now fairly well established that radiation has profound effects on vascular function in the irradiated tissue, resulting in alterations in blood flow (Zanelli & Lucas, 1976), in the structure of the capillary endothelium (Eddy, 1980) and in the extravasation of plasma macromolecules (Krishnan *et al.*, 1988). Furthermore, there are indications that the vascular effects of radiation are not random but systematic, i.e. are functions of the radiation dose and the time after radiation, and that the sequence of events is different in normal and malignant tissues (Zanelli & Lucas, 1976). Although the effects of radiation on cell membranes are less well understood they appear to involve loss of surface lipids (Bacq & Alexander, 1966) leading to loss of integrity and altered permeability to a variety of chemical entities.

One further point of importance in the present context concerns the shape of the cell survival curves following chemotherapy. Although much is known about cell survival curves after irradiation, we know much less about cell survival after chemotherapy. The available evidence (Masters & Hepburn, 1986) suggests that biphasic or exponential cell survival curves with very different slopes can be obtained depending on the choice of drug and the cell line under investigation. A judicious choice of drug/cell line combination can result in exponential cell survival curves with steep slopes. In this case doubling the amount of drug reaching the target cells can lead to much more than double the effect on the cell population. On the other hand, if the amount of drug reaching the cellular milieu is reduced (e.g. due to the physiological effects of radiation on the vasculature) then the effects of chemotherapy may be substantially reduced.

There are at present no data at all on the effects of radiation on tumour levels of chemotherapeutic agents during fractionated radiotherapy. Plasma levels of drugs are not much help since it has been shown that they may bear little or no relationship to tumour levels (Donelli *et al.*, 1984; Anderson *et al.*, 1970), yet most reports on new regimes of combined radiotherapy–chemotherapy confine themselves to reporting plasma levels and urinary excretion of the drugs. In what follows, a systematic approach has been devised to assess the quantity of a widely (and commercially available in radioactively labelled form) used chemotherapeutic agent (vincristine) which accumulates in an experimental mouse tumour model throughout a realistic course of fractionated radiotherapy.

### Materials and methods

### *Mice and tumour*

Eight- to twelve-week-old male CBA/He mice were used throughout. They were kept under standard animal house conditions; controlled light cycles, and allowed access to food and water *ad libitum*.

The tumour was a carcinoma, designated as carcinoma NT or neck tumour, which arose spontaneously in this strain of mice some 20 years ago and has been serially passaged ever since. This tumour, which has been fully described by Hewitt *et al.* (1976), has now been transplanted into more than 10,000 mice. There has never been a single 'no take' or spontaneous regression.

For transplanting, tumour tissue was minced with fine, sterilised scissors in a sterile Petri dish. The mince was then transferred to a sterile tube, shaken with 5–10 ml of saline and allowed to stand for a few minutes until all the coarse debris had settled. After a quick cell count in a haemocytometer chamber, the volume of supernatant was adjusted to contain a concentration of $15–20 \times 10^3$ cells in an injection volume of 0.05 ml and this volume was injected intradermally in the previously shaven flank of recipient mice using a 1 ml syringe and 25 G needle.

During all experiments the tumours were measured with calipers in three perpendicular dimensions. A mean geometrical diameter was calculated as $d = (abc)^{1/3}$.

Correspondence: G.D. Zanelli.
Received 3 February 1989, and in revised form, 6 April 1989.

*Irradiation*

The available source was a 3 kCi cobalt-60 small animal irradiation facility. The heavy field-defining cone of the irradiator was used to protect the mice's bodies. The dose rate at the site of the tumour and the body was measured with an ionization chamber and lithium fluoride thermoluminescence dosimetry. The dose rate at the beginning of the series of experiments was 10.2 cGy $min^{-1}$ at the tumour site and 0.006 cGy $min^{-1}$ at the centre of a mouse's body. During the course of the experiments the dose-rate was corrected weekly for the decay of the source. The irradiation chamber was kept at about 30°C during irradiation to prevent hypothermia in the mice which were sedated with mild doses of Hypnorm i.p. (Jansen Pharmaceutical Ltd, Oxford, UK).

*$^{3}H$-vincristine*

Tritiated vincristine sulphate was bought from Amersham International (Amersham, Bucks, UK, code TRK 478, sp. act 74–370 GBq $mol^{-1}$). It is supplied in 0.2 ml of 0.01 N sulphuric acid and for injection it was diluted with saline to an activity content of about 110 kBq in 0.1 ml and this volume was injected i.v. in a lateral tail vein of the mice. The amount of vincristine injected varied between 0.18 and 2.3 μg.

*Experimental protocols*

The project reported herein involved six separate but interdependent experiments. In general, a larger number of mice than that required for any given experiment were inoculated intradermally with $15–20 \times 10^3$ tumour cells. Out of these the required number of animals was selected on the basis of similar tumour size and true intradermal site. Irradiation was always started when the tumours were 5–6 mm in average diameter. For the sake of clarity each experiment will be described individually.

*Experiment 1* One hundred tumour bearing mice were started on daily treatments with 2 Gy of radiation, five days per week for five weeks, beginning on a Monday. Irradiation was always carried out between 8.30 and 9.30 in the morning. After each fraction, four mice were withdrawn from the experiment, injected with $^{3}H$-vincristine i.v. 1 h after the midpoint of the radiation treatment and killed (stunning and decapitation) 1 h after the injection of the tracer. The tumour, a blood sample (from the severed neck vessels) and various organs (liver, spleen, kidneys, lungs) were removed and accurately weighed. For this and all subsequent experiments the $^{3}H$ content of the tissues was measured by burning them in a sample oxidiser (Packard Sample Oxydizer, model B306, Packard Instruments, Caversham, UK) followed by counting in an LKB Wallac 1280 gamma counter (Pharmacia Ltd, Milton Keynes, UK) together with appropriate standards (aliquots of the injected dose burned as for the tissues).

*Experiment 2* The tumours of 24 mice were given $7 \times 2$ Gy daily irradiation. The mice were then injected in groups of four with $^{3}H$-vincristine at various times later (0.5–24 h) and killed 1 h after injection.

*Experiment 3* A group of 24 mice had their tumours irradiated to $7 \times 2$ Gy. The mice were injected with the tracer 4 h after the last fraction and killed in groups of four at various times (0.5–24 h) after injection.

*Experiment 4* Since the tumours continue to grow during a protracted regime of fractionated irradiation, the effect of tumour size on the uptake of $^{3}H$-vincristine per unit weight of tumour tissue had to be determined. In experiment 4 therefore a large group of mice was inoculated intradermally with the tumour cells and their tumours measured daily. During the growth of the tumours (~4 weeks) groups of four mice with tumours of similar size were injected with the tracer, killed 1 h later and the $^{3}H$ content per unit weight of tumour determined as described above. This was continued until tumours of a mean diameter of ~12 mm were obtained and the experiment was then stopped.

*Experiment 5* Eight groups of four mice with non-irradiated tumours of similar size ($d = 5.8 \pm 0.6$ mm) were injected with $^{3}H$-vincristine i.v. and killed at various times (0.5–24 h) after injection. The tumour and blood content of $^{3}H$ was determined as usual.

*Experiment 6* This was the same as experiment 1 (daily irradiations of 2 Gy, five days per week for 5 weeks) except that after each fraction a group of four mice was withdrawn and injected 4 h after irradiation (1 h in experiment 1) and killed 1 h after injection (as for experiment 1). In this experiment only the tumour and blood were collected.

### Results

The results of experiment 1 are shown in Figure 1. There are substantial variations in the uptake of $^{3}H$ per unit weight of tumour throughout the course of fractionated radiation. After $7 \times 2$ Gy the amount of tracer in the tumour is more than twice that found at the beginning of the experiment. On the other hand, after 15–18 fractions of 2 Gy the uptake falls to about one-third of that on day 1. In this and all subsequent figures the bars represent the standard error of the means.

The troughs at 9 and 17 days could be due to a reduction in uptake in larger tumours as shown in Figure 2, coupled with recovery of the vasculature during the week-end rest

**Figure 1** Experiment 1: $^{3}H$ content per gram of tumour as percentage of the injected dose after each of 25 daily fractions of 2 Gy $^{60}Co$ irradiation to the tumour. The mice were injected with $^{3}H$-vincristine i.v. 1 h after irradiation and killed 1 h after injection. The vertical bars are the standard errors of the means ($n = 4$).

**Figure 2** Effect of tumour size ($d = (abc)^{1/3}$) on the uptake of $^{3}H$. The mice were killed and the tumours excised one hour after the injection (i.v.) of $^{3}H$-vincristine.

period from radiation damage. The peaks appear to be real and suggest increased extravasation of drug through radiation damaged capillary endothelium.

Figure 3 shows the percentage of injected dose per gram of liver and lung throughout the course of fractionated tumour irradiation in experiment 1. The uptake in these organs is fairly constant. The growth pattern of the NT tumour with and without irradiation is shown in Figure 4. This tumour is fairly radioresistant and continues to grow, at a reduced rate, during the radiation treatment (the data points are for a group of mice which had undergone the full 5 week treatment during experiment 1). There is little evidence of tumour control even after 25 fractions of 2 Gy.

Figure 5 shows that in non-irradiated tumours the uptake of $^{3}H$ per unit weight remains reasonably constant up to 24 h after injection (experiment 5). The amount of tracer in the tumour tissue stays below the concentration in the blood.

Figures 6 and 7 show the pattern of uptake of $^{3}H$ in tumours given seven fractions of 2 Gy (the peak of uptake in experiment 1, Figure 1) with time after irradiation (experiment 2, Figure 6) and time after injection (experiment 3, Figure 7). They show that the maximum uptake in tumours is achieved if the mice are injected four hours after irradiation and killed one hour after injection. Furthermore, the amount of tracer in the tumours after 7 × 2 Gy of

**Figure 3** $^{3}H$ content per gram of liver (filled circles) and lung (open circles) tissue during a fractionated course of 2 × 25 Gy $^{60}Co$ irradiation delivered to the tumour only. The vertical bars are the standard errors of the means ($n$ = 4).

**Figure 4** Growth of the carcinoma-NT. Open circles, non-irradiated controls; filled circles, tumours given 2 Gy daily $^{60}Co$-irradiation.

**Figure 5** $^{3}H$ content per gram of tumour and blood of non-irradiated mice as a function of time after i.v. injection of $^{3}H$-vincristine. The vertical bars are the standard errors of the means ($n$ = 4). Open circles, blood; filled circles, tumour.

**Figure 6** $^{3}H$ content per gram of tissue in mice given 7 × 2 Gy daily $^{60}Co$ irradiation to the tumour, injected with $^{3}H$-vincristine i.v. at various times after the last fraction and killed 1 h after injection. Filled circles, tumour; open circles, blood. The vertical bars are the standard errors of the means ($n$ = 4).

radiation is greater than that in non-irradiated tumours (Figure 5) and is above the blood concentration.

Figure 8 shows the results of experiment 6 in which the experimental conditions have been optimised on the basis of the results of experiments 2 and 3, i.e. the mice were injected 4 h after a given fraction of radiation and killed one hour after injection. The rhythmic pattern of tracer uptake is similar to that of experiment 1 (Figure 1) but the absolute values are higher, as would be expected from the results shown in Figures 6 and 7.

**Figure 7** $^{3}H$ content per gram of blood and tumour in mice given 7 × 2 Gy daily $^{60}Co$ irradiation to the tumour, injected i.v. with $^{3}H$-vincristine 1 h after the last fraction and killed at various times after injection. Filled circles, tumour; open circles, blood. The vertical bars are the standard errors of the means ($n$=4).

**Figure 8** Experiment 6: $^{3}H$ content per gram of tumour as percentage of the injected dose after each of 25 daily fractions of 2 Gy $^{60}Co$ irradiation to the tumour. The mice were injected with $^{3}H$-vincristine i.v. 4 h after irradiation and killed 1 h after injection. The vertical bars are the standard errors of the means ($n$=4).

### Discussion

Combined radiotherapy and chemotherapy in the treatment of human cancer is slowly gaining favour. Early in the use of CMT it became apparent that radiation and drugs could interact in several ways and attempts were made to rationalise the semantics of such interactions (Steel, 1979), to study their mechanisms (Wheeler & Kaufman, 1980; van der Maase, 1984; Joiner *et al.*, 1984) and to propose more realistic models for the study of such interactions in humans (Rubin *et al.*, 1988).

It has recently become evident that the time sequence of administration of the two modalities plays an important part in determining the observed results (Twentyman *et al.*, 1979; Begg *et al.*, 1979; Collis & Steel, 1983; Pearson & Steel, 1984; Fu *et al.*, 1985, 1988; Lelieveld *et al.*, 1985). Although in most cases involving experimental mouse tumours single doses, continuous low dose-rate or a small number of large fractions of radiation were used, the results in general tend to show that there is greater enhancement of the radiation effects when the drugs are given shortly before or shortly after the radiation. Depending on the effects observed the results have been variously interpreted as due to factors such as repopulation, movement of cells to a phase more sensitive to the drug or inhibition of recovery from radiation damage. Although these factors are very likely to play a role in the observed results it is difficult to draw any firm conclusions unless the actual quantity of drug reaching the target cells is known with reasonable accuracy: lack of potentiation may simply mean that only a small percentage of the drug reaches or is prevented from reaching the tumour tissues. That this can happen during a realistic course of fractionated radiation has been shown in this communication and has been recognised as a possible source of error in CMT by other authors (Looney *et al.*, 1985; Tannock & Sindelar, 1988).

As for possible explanations of the results reported in this paper, the effects of radiation on the tumour vasculature must be the prime candidate. It has been known for a long time that radiation has severe effects on blood flow and extravasation of macromolecules (Zanelli & Lucas, 1976; Song & Levitt, 1970) and has recently been confirmed by Krishnan *et al.*, (1988), who showed that there is an immediate increase in extravasation of albumin after doses of radiation as low as 2 Gy.

The sinusoidal character of the uptake curves (Figures 1 and 8) is a hitherto unexplained but reproducible phenomenon. Zanelli (1977) has shown that the blood pulse in irradiated human skin during and after a course of fractionated radiotherapy shows similar variations in time. As far back as 1928, Mottram showed that after irradiation tumour cells swell up and tend to occlude the capillaries. It is conceivable that during fractionated radiation periods of cellular swelling and oedema alternate with periods of decreased cellularity and reopening of the blood vessels. The fact that the peaks of uptake (Figures 1 and 8) seem to coincide with the days following the week-end rest also suggests that the 'wave like' effects seen could be due to some mechanism of recovery from radiation damage. This opens up interesting possibilities in view of current thoughts of continuing radiotherapy over week-ends.

Whether the results of the present investigation apply to all drugs and all tumours, especially human tumours, is not known, and neither is the effect of different fractionation regimes. On the basis of the above discussion one would expect all drugs to behave at least qualitatively in a similar manner. However, different tumours and fractionation schedules may show different temporal effects.

As stated in the Introduction the available evidence suggests that the effects of radiation on malignant and normal tissues with respect to extravasation are qualitatively similar but displaced in time. If one has to calculate parameters such as 'therapeutic gain' from CMT experiments it is essential that these temporal differences be investigated during realistic regimes of fractionated radiation, i.e. with experiments of the type reported above on tumours and normal tissues.

Since in clinical practice concurrent radiotherapy and chemotherapy are not only being seriously considered, but actually practised, the above questions are critical. Animal experiments are obviously the quickest way of obtaining answers, but the question still remains of how closely animal models mimic the human situation. The type of experiments described in this paper can be carried out in some types of human cancer where at least one extra biopsy can be taken without undue discomfort to the patients. Alternatively, with modern non-invasive methods of detection (e.g. positron emission tomography) it may be possible to quantitate tissue uptake of therapeutic agents labelled with positron-emitting radionuclides.

The authors would like to thank the Italian Cancer Research Society 'La via di Natale' for their generous support.

### References

ANDERSON, L.L., COLLINS, G.J., OJIMA, Y. & SULLIVAN, R.D. (1970). A study of the distribution of Methotrexate in human tissues and tumours. *Cancer Res.*, **30**, 1344.

BACQ, Z.M. & ALEXANDER, P. (1966). *Fundamentals of Radiobiology*. Pergamon Press: Oxford.

BEGG, A.C., FU, K.K. & PHILLIPS, T.L. (1979). Combination therapy of a solid murine tumour with cyclophosphamide and radiation: the effects of time, dose and assay method. *Int. J Radiat. Oncol. Biol. Phys.*, **5**, 1433.

BROWN, J.M. (1979). Drug or radiation changes to the host which could affect the outcome of combined modality therapy. *Int. J. Radiat. Oncol. Biol. Phys.*, **5**, 1151.

COLLIS, C.H. & STEEL, G.G. (1983). Lung damage in mice from cyclophosphamide and thoracic irradiation: the effect of timing. *Int. J. Radiat. Oncol. Biol. Phys.*, **9**, 685.

DONELLI, M.G., D'INCALCI, M. & GARATTINI, S. (1984). Pharmacokinetic studies of anticancer drugs in tumour-bearing animals. *Cancer Treatment Rep.*, **68**, 381.

EDDY, H.A. (1980). Tumour vascular response following irradiation. *Microvasc. Res.*, **20**, 195.

FU, K.K., LAM, K.N. & RAYNE, P.A. (1985). The influence of time sequence of cisplatin administration and continuous low dose rate irradiation (CLDRI) on their combined effects on a murine squamous cell carcinoma. *Int. J. Radiat. Oncol. Biol. Phys.*, **44**, 2113.

FU, K.K., DE GREGORIO, M.W. & PHILLIPS, J.W. (1988). Plasma and tumour concentrations of cisplatin following intraperitoneal infusion or bolus injection with or without continuous low-doserate irradiation. NCI Monographs, Conference on the Interaction of Radiation Therapy and Chemotherapy, no. 6, 123.

HEWITT, H.B., BLAKE, E.R. & WALDER, A.S. (1976). A critique of the evidence for the active host defence against cancer, based on personal studies of 27 murine tumours of spontaneous origin. *Br. J. Cancer*, **23**, 241.

HOWES, A.E. (1988). Keynote address: Models of normal tissue injury following combined modality therapy. NCI Monographs, Conference on the Interaction of Radiation Therapy and Chemotherapy, no. 6, 5.

JOINER, M.C., BREMNER, J.C.M. & DENEKAMP, J. (1984). The therapeutic advantage of combined x-rays and melphalan. *Int. J. Radiat. Oncol. Biol. Phys.*, **10**, 385.

KRISHNAN, L., KRISHNAN, E.C. & JEWELL, W.R. (1988). Immediate effect of irradiation on microvasculature. *Int. J. Radiat. Oncol. Biol. Phys.*, **15**, 147.

LELIEVELD, P., SCOLES, M.A., BROWN, J.M. & KALLMAN, R.F. (1985). The effect of treatment in fractionated schedules with the combination of pre-irradiation and six drugs on the RIF-1 tumour and normal mouse skin. *Int. J. Radiat. Oncol. Biol. Phys.*, **11**, 111.

LOONEY, W.B., HOPKINS, H.A. & CARTER, W.H. (1985). Solid tumour models for the assessment of different treatment modalities. XXIII. A new approach to more effective utilization of radiotherapy alternated with chemotherapy. *Int. J. Radiat. Oncol. Biol. Phys.*, **11**, 2105.

MASTERS, J.R.W. & HEPBURN, P.J. (1986). Human bladder cancer *in vitro* drug sensitivities: range and stability in long term culture. *Br. J. Cancer*, **54**, 131.

MOTTRAM, J.C. (1928). Action of radiation on blood supply of tumours. *Lancet*, **ii**, 966.

PEARSON, A.E. & STEEL, G.G. (1984). Chemotherapy in combination with pelvic irradiation: a time-dependent study. *Radiother. Oncol.*, **2**, 49.

RUBIN, P., CONSTINE, L.S. & VAN ESS, J.D. (1988). Special lecture: Scoring of late toxic effects – interaction of two modalities. NCI Monographs, Conference on the Interaction of Radiation Therapy and Chemotherapy, no. 6, 9.

SONG, C.W. & LEVITT, S.H. (1970). Effect of x-irradiation on vascularity of normal tissue and experimental tumour. *Radiology*, **44**, 445.

STEEL, G.G. (1979). Terminology in the description of drug-radiation interactions. *Int. J. Radiat. Oncol. Biol. Phys.*, **5**, 1145.

STEEL, G.G. (1988). The search for therapeutic gain in the combination of radiotherapy and chemotherapy. *Radiother. Oncol.*, **11**, 31.

TANNOCK, I.F. & SINDELAR, W.F. (1988). Discussion summary. NCI Monographs, Conference on the Interaction of Radiation Therapy and Chemotherapy, no. 6, 155.

TUBIANA, M., ARRIAGADA, R. & COSSET, J.M. (1985). Sequencing of drugs and radiation. *Cancer*, **55**, 2131.

TWENTYMAN, P.R., KALLMAN, R.F. & BROWN, J.M. (1979). The effect of time between re-irradiation and chemotherapy on the growth of three solid mouse tumours – II. Cyclophosphamide. *Int. J. Radiat. Oncol. Biol. Phys.*, **5**, 1425.

VAN DER MAASE, H. (1984). Interaction of radiation and adriamycin, bleomycin, mitomycin C or cisdiammine dichloroplatinum II in intestinal crypt cells. *Br. J. Cancer*, **49**, 779.

WHEELER, K.T. & KAUFMAN, K. (1980). Influence of fractionation schedules on the response of a rat brain tumour to therapy with BCNU and radiation. *Int. J. Radiat. Oncol. Biol. Phys.*, **6**, 845.

ZANELLI, G.D. & LUCAS, P.B. (1976). Effects of x-rays on vascular function in transplanted tumours and normal tissues in the mouse. *Br. J. Cancer*, **34**, 408.

ZANELLI, G.D. (1977). The effects of radiation on the skin blood volume pulse in humans. *Br. J. Radiol.*, **50**, 68.


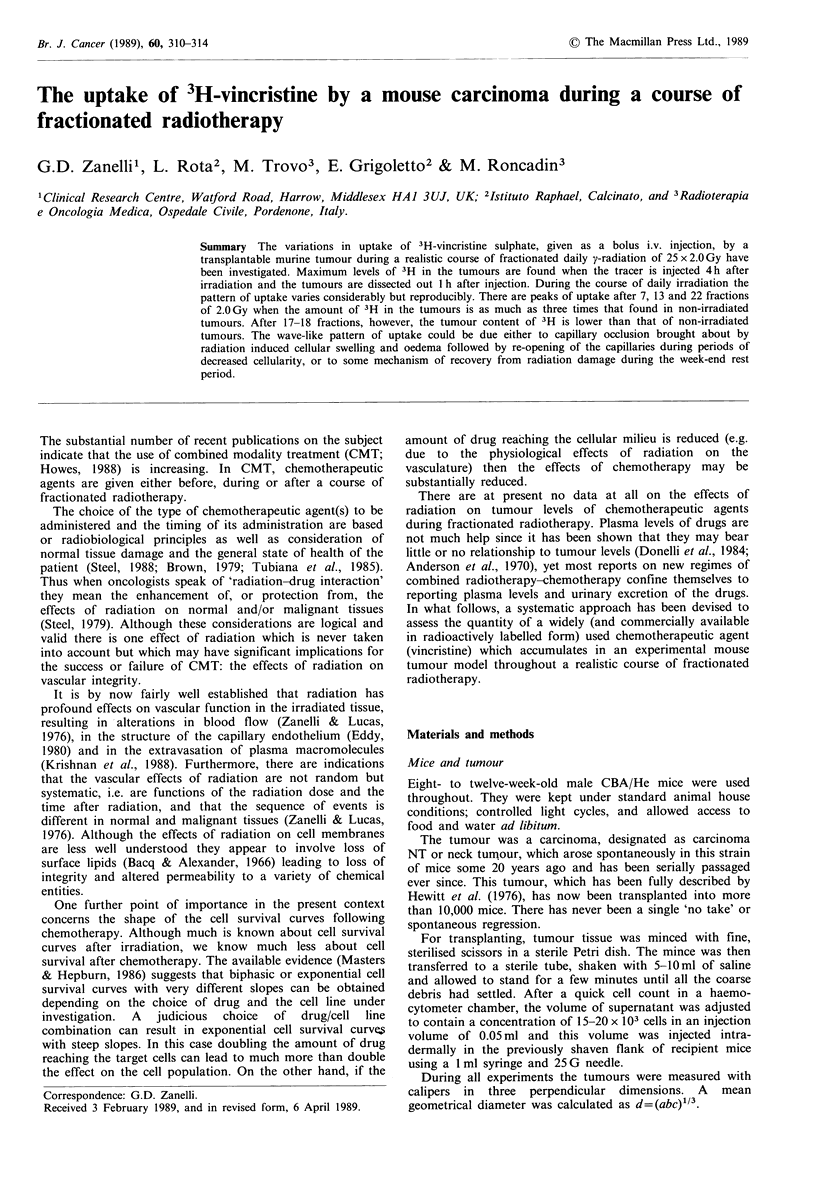

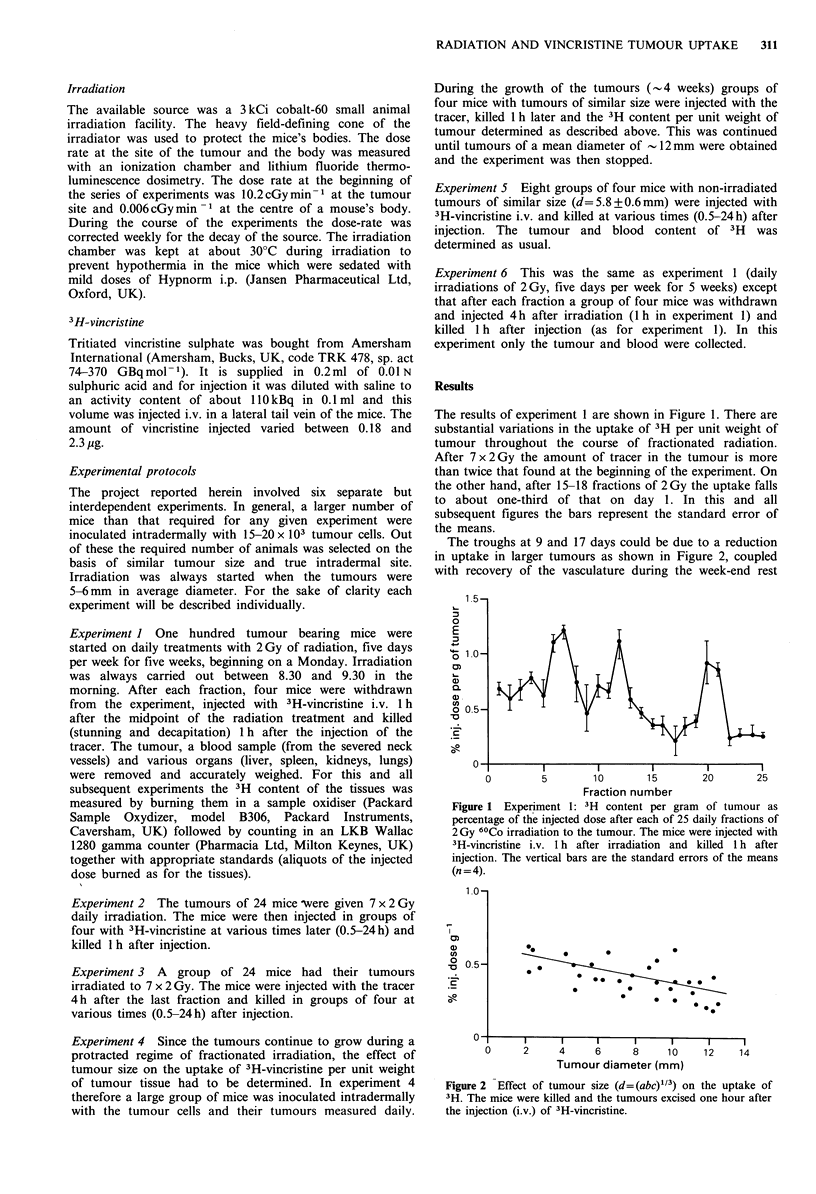

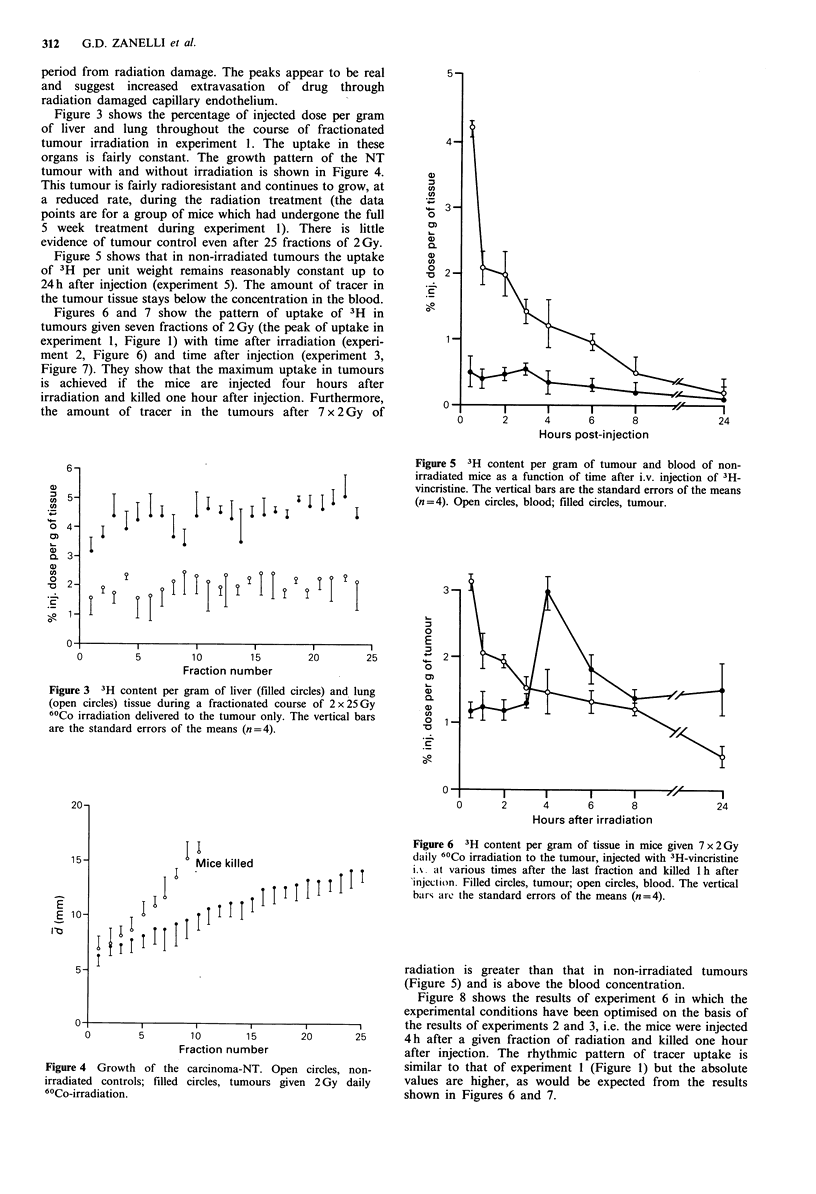

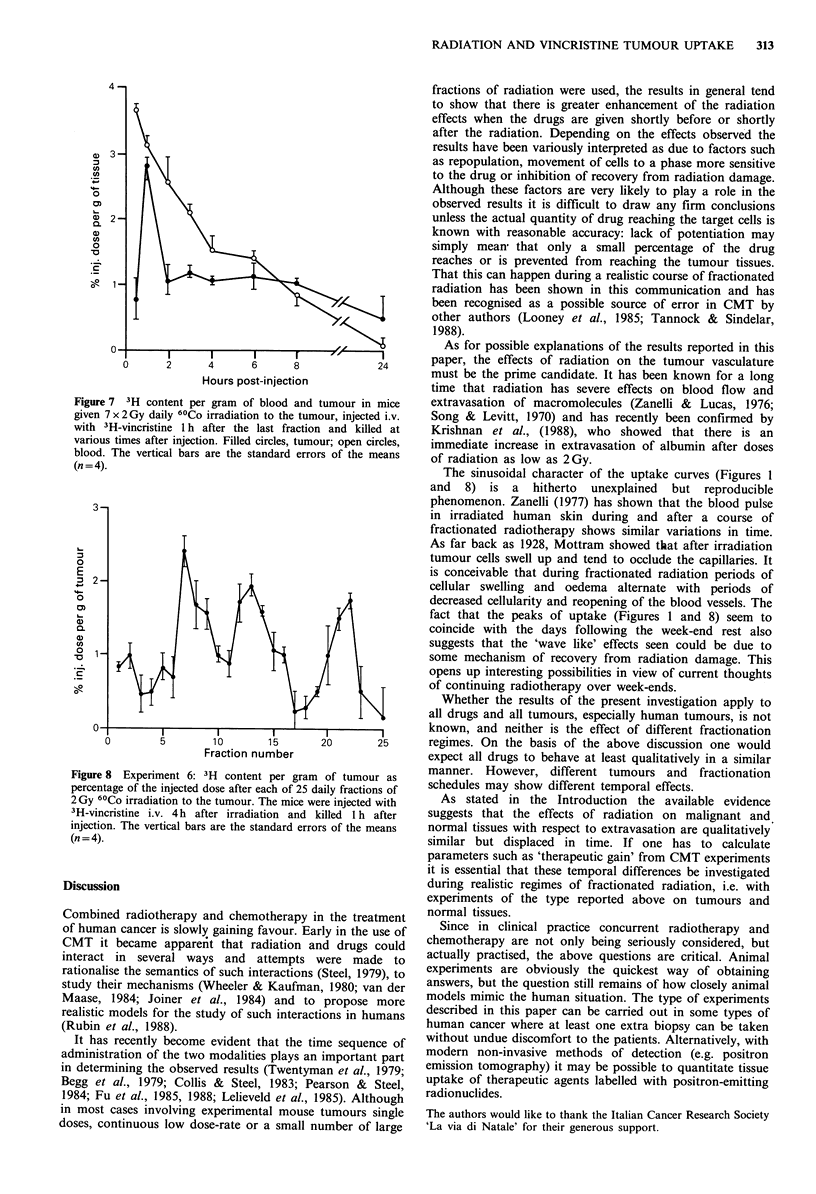

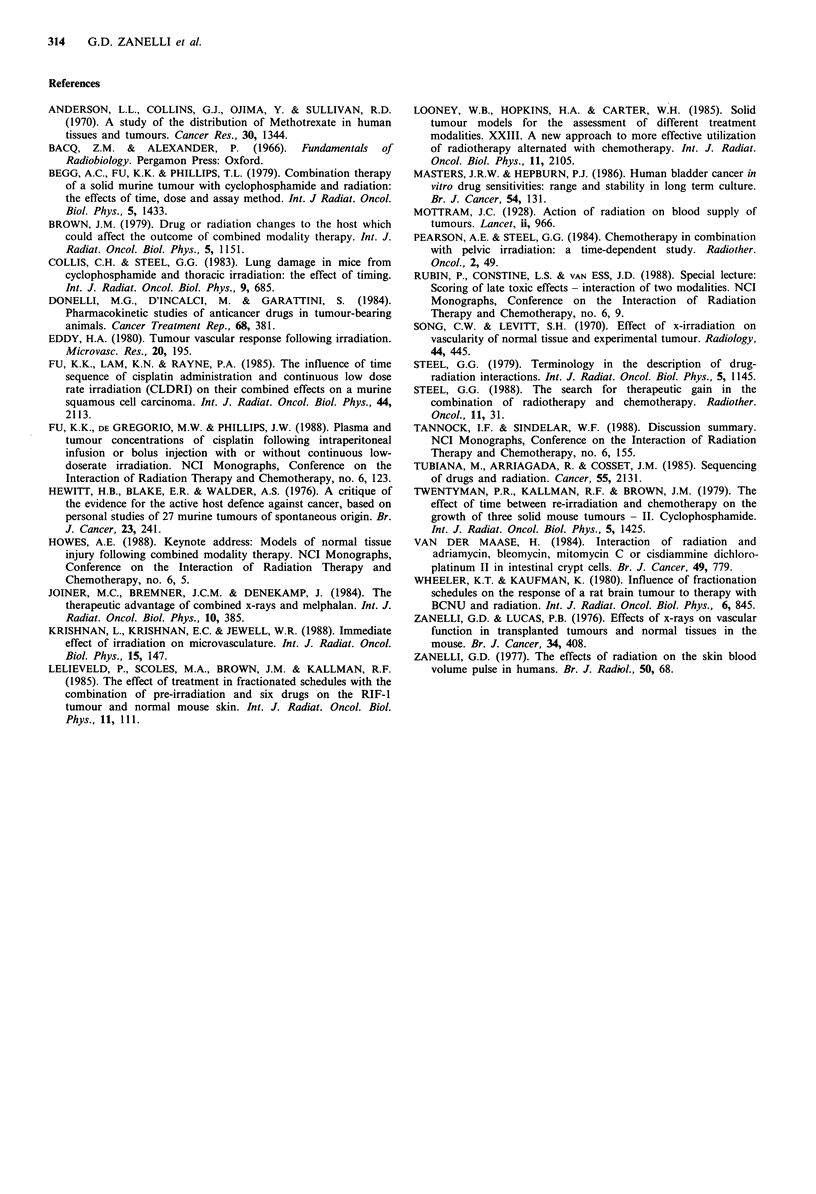


## References

[OCR_00719] Anderson L. L., Collins G. J., Ojima Y., Sullivan R. D. (1970). A study of the distribution of methotrexate in human tissues and tumors.. Cancer Res.

[OCR_00728] Begg A. C., Fu K. K., Shrieve D. C., Phillips T. L. (1979). Combination therapy of a solid murine tumor with cyclophosphamide and radiation: the effects of time, dose and assay method.. Int J Radiat Oncol Biol Phys.

[OCR_00734] Brown J. M. (1979). Drug or radiation changes to the host which could affect the outcome of combined modality therapy.. Int J Radiat Oncol Biol Phys.

[OCR_00739] Collis C. H., Steel G. G. (1983). Lung damage in mice from cyclophosphamide and thoracic irradiation: the effect of timing.. Int J Radiat Oncol Biol Phys.

[OCR_00744] Donelli M. G., D'Incalci M., Garattini S. (1984). Pharmacokinetic studies of anticancer drugs in tumor-bearing animals.. Cancer Treat Rep.

[OCR_00749] Eddy H. A. (1980). Tumor vascular responses following irradiation.. Microvasc Res.

[OCR_00760] Fu K. K., DeGregorio M. W., Phillips J. W. (1988). Plasma and tumor concentrations of cisplatin following intraperitoneal infusion or bolus injection with or without continuous low-dose-rate irradiation.. NCI Monogr.

[OCR_00766] Hewitt H. B., Blake E. R., Walder A. S. (1976). A critique of the evidence for active host defence against cancer, based on personal studies of 27 murine tumours of spontaneous origin.. Br J Cancer.

[OCR_00778] Joiner M. C., Bremner J. C., Denekamp J. (1984). The therapeutic advantage of combined X-rays and melphalan.. Int J Radiat Oncol Biol Phys.

[OCR_00783] Krishnan L., Krishnan E. C., Jewell W. R. (1988). Immediate effect of irradiation on microvasculature.. Int J Radiat Oncol Biol Phys.

[OCR_00788] Lelieveld P., Scoles M. A., Brown J. M., Kallman R. F. (1985). The effect of treatment in fractionated schedules with the combination of X-irradiation and six cytotoxic drugs on the RIF-1 tumor and normal mouse skin.. Int J Radiat Oncol Biol Phys.

[OCR_00795] Looney W. B., Hopkins H. A., Carter W. H. (1985). Solid tumor models for the assessment of different treatment modalities: XXIII. A new approach to the more effective utilization of radiotherapy alternated with chemotherapy.. Int J Radiat Oncol Biol Phys.

[OCR_00802] Masters J. R., Hepburn P. J. (1986). Human bladder cancer in vitro drug sensitivities: range and stability in long-term culture.. Br J Cancer.

[OCR_00811] Pearson A. E., Steel G. G. (1984). Chemotherapy in combination with pelvic irradiation: a time-dependence study in mice.. Radiother Oncol.

[OCR_00822] Song C. W., Levitt S. H. (1970). Effect of x irradiation on vascularity of normal tissues and experimental tumor.. Radiology.

[OCR_00827] Steel G. G. (1979). Terminology in the description of drug-radiation interactions.. Int J Radiat Oncol Biol Phys.

[OCR_00830] Steel G. G. (1988). The search for therapeutic gain in the combination of radiotherapy and chemotherapy.. Radiother Oncol.

[OCR_00840] Tubiana M., Arriagada R., Cosset J. M. (1985). Sequencing of drugs and radiation. The integrated alternating regimen.. Cancer.

[OCR_00844] Twentyman P. R., Kallman R. F., Brown J. M. (1979). The effect of time between X-irradiation and chemotherapy on the growth of three solid mouse tumors--II. Cyclophosphamide.. Int J Radiat Oncol Biol Phys.

[OCR_00855] Wheeler K. T., Kaufman K. (1980). Influence of fractionation schedules on the response of a rat brain tumor to therapy with BCNU and radiation.. Int J Radiat Oncol Biol Phys.

[OCR_00859] Zanelli G. D., Lucas P. B. (1976). Effects of X-rays on vascular function in transplanted tumours and normal tissues in the mouse.. Br J Cancer.

[OCR_00864] Zanelli G. D. (1977). The effects of radiation on the skin blood volume pulse in humans.. Br J Radiol.

[OCR_00850] von der Maase H. (1984). Interactions of radiation and adriamycin, bleomycin, mitomycin C or cis-diamminedichloroplatinum II in intestinal crypt cells.. Br J Cancer.

